# Homozygous *GRID2* missense mutation predicts a shift in the D-serine binding domain of GluD2 in a case with generalized brain atrophy and unusual clinical features

**DOI:** 10.1186/s12881-017-0504-6

**Published:** 2017-12-06

**Authors:** Zafar Ali, Shumaila Zulfiqar, Joakim Klar, Johan Wikström, Farid Ullah, Ayaz Khan, Uzma Abdullah, Shahid Baig, Niklas Dahl

**Affiliations:** 1 Department of Immunology, Genetics and Pathology, Science for Life Laboratory, Uppsala University, BMC Box815, 751 08 Uppsala, Sweden; 2 Human Molecular Genetics Laboratory, National Institute for Biotechnology and Genetic Engineering (NIBGE), PIEAS, Faisalabad, 38000 Pakistan; 3 Department of Radiology, Uppsala University, 751 85 Uppsala, Sweden

**Keywords:** Cerebellar syndrome, Cerebral atrophy, Developmental delay, *GRID2* gene, GluD2, Whole exome sequencing, Mutation, 3D protein modeling

## Abstract

**Background:**

Spinocerebellar ataxias comprise a large and heterogeneous group of disorders that may present with isolated ataxia, or ataxia in combination with other neurologic or non-neurologic symptoms. Monoallelic or biallelic *GRID2* mutations were recently reported in rare cases with cerebellar syndrome and variable degree of ataxia, ocular symptoms, hypotonia and developmental delay.

**Case presentation:**

We report on a consanguineous family with autosomal recessive childhood onset of slowly progressive cerebellar ataxia and delayed psychomotor development in three siblings. MRI of an adult and affected family member revealed slightly widened cerebral and cerebellar sulci, suggesting generalized brain atrophy, and mild cerebellar atrophy. Using whole exome sequencing we identified a novel homozygous missense variant [c.2128C > T, p.(Arg710Trp)] in *GRID2* that segregates with the disease. The missense variant is located in a conserved region encoding the extracellular serine-binding domain of the GluD2 protein and predicts a change in conformation of the protein.

**Conclusion:**

The widespread supratentorial brain abnormalities, absence of oculomotor symptoms, increased peripheral muscle tone and the novel missense mutation add to the clinical and genetic variability in *GRID2* associated cerebellar syndrome. The neuroradiological findings in our family indicate a generalized neurodegenerative process to be taken into account in other families segregating complex clinical features and *GRID2* mutations.

## Background

Early onset autosomal recessive spinocerebellar ataxias constitute a heterogeneous group of mostly progressive disorders [1]. To date more than 40 different spinocerebellar ataxia (SCA) types are described with an extensive genetic heterogeneity and with all modes of transmissions represented [2]. Typical clinical features are ataxia with loss of gait and limb coordination, dysarthria, sometimes accompanied with altered ocular movements. The clinical expression is variable between, as well as within the different clinical entities and may include cognitive decline and intellectual disability (ID). Recently, deletions involving the *GRID2* gene, encoding the glutamate receptor subunit delta-2 (GluD2) protein, were reported in families segregating autosomal recessive cerebellar syndrome with infantile onset [3–5]. Affected individuals showed slowly progressive hypotonia, developmental delay, abnormal eye movements, ataxia and dysarthria. Radiological investigation revealed cerebellar atrophy, in a few cases with pontine involvment. Furthermore, heterozygous *GRID2* mutations, including three missense variants, have been identified in rare cases with variable onset of cerebellar ataxia, ocular symptoms and cognitive impairment [6, 7].

In this study, we performed exome sequencing in a consanguineous Pakistani family segregating autosomal recessive early-onset cerebellar syndrome and global developmental delay in three affected siblings. The combined clinical, radiological and genetic findings add further genotype-phenotype correlations to *GRID2* associated ataxia.

## Case presentation

**Fig. 1:**
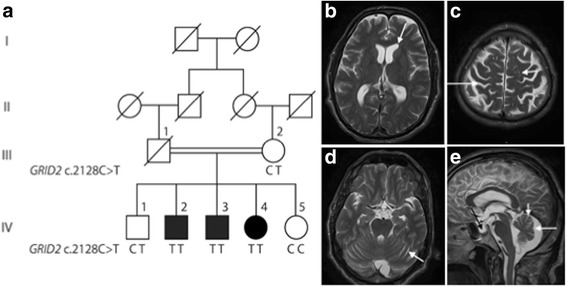
Pedgree, genotypes and MRI of the consanguineous family segregating the *GRID2* variant (**a**) Pedigree and genotypes of the consanguineous family segregating *GRID2* mutation c.2128C > T, p.(Arg710Trp). (**b**) MRI findings in ind. V:2 showing slight to moderate widening of cerebral ventricles (arrow). (**c**) Slight to moderate widening of cerebral sulci (long arrow) and prominent perivascular spaces (short arrow). (**d**) Slightly widened cerebellar sulci (short white arrow). (**e**) Widened cerebellar sulci (short white arrow). The retrocerebellar arachnoid cyst with slight compression of the vermis (long white arrow) and some degree of intrasellar cisternal herniation (black arrow) are likely incidental findings. The combined MRI findings indicate a slight and general brain atrophy without involvement of the brainstem. Transverse (**b**-**d**) and sagittal (**e**) T2-weighted images. Images were obtained on a 1.5 T scanner, and included sagittal T1- and T2-weighted and axial T2-weighted images with a slice thickness of 5 mm. The abnormalities were assessed qualitatively and based on age related reference subjects [8]

We identified a consanguineous Pakistani family segregating congenital or infantile onset of autosomal recessive SCA with developmental delay and moderate intellectual disability in three siblings (Fig. 1a). Family history revealed that the affected individuals produced their first words at ages 3-4 years and learned to walk without assisted ambulation at ages 7-8 years. The delay was accompanied by increased and stationary peripheral muscle tone since childhood. As adults, the affected siblings speak with 3-4 words sentences, often without logic, using a total vocabulary of approximately 100 words. They required help with self-care such as dressing, feeding and self-cleaning. Examination of the three affected and two unaffected siblings as well as their mother was performed by a neurologist. The main clinical features of the family members investigated at adult age are summarized in Table 1. The examination revealed increased peripheral muscle tone in the three affected siblings whereas ocular symptoms (nystagmus, upgaze) were absent. Visual acuity was normal but eye fundus examination was not possible to perform. Based on the structured interview and the clinical investigation, the cognitive impairment was considered stationary from adolescence equivalent to moderate intellectual disability.

**Table 1:** Clinical features of family members

	Individual						
	III:1^a^	III:2	IV:1	IV:2	IV:3	IV:4	IV:5
*GRID2* genotype	NI	C/T	C/T	T/T	T/T	T/T	C/C
Gender	M	F	M	M	M	F	F
Age (years) at exam	† at 70	75	53	47	45	40	35
Age at onset (ataxia)	–	–	–	Congenital	Congenital	Congenital	–
Cognitive impairment	–	–	–	Moderate intellectual disability	Moderate intellectual disability	Moderate intellectual disability	–
Atactic gait	–	–	–	+	+	+	–
Dysmetria	–	–	–	+	+	+	–
Dysdiadochokinesis	NI	–	–	+	+	+	–
Dysarthria	–	–	–	+	+	+	–
Peripheral muscle tone	Normal	Normal	Normal	Increased	Increased	Increased	–
Nystagmus upgaze/ saccadic pursuit	NI	–	–	–	–	–	–
Ambulation	Normal	Normal	Normal	Walk independently from age 7-8 years	Walk independently from age 7-8 years	Walk independently from age 7-8 years	Normal
MRI findings	NI	NI	NI	General atrophy	NI	NI	NI
Babinski sign	NI	–	–	–	–	–	–
Delayed motor development	–	–	–	+	+	+	–
Body length (cm)	NI	149	171	172	162	152	150
Head circumference (cm)	NI	55	55.5	56	55.5	53	53.5

+ = present; − = absent;; NI = not investigated, F = female, M = male, † = deceased

^a^Ind. III:1 died at age 70 years and his clinical data are based on anamnestic information from III:2

One affected individual (IV:2) was available for MRI investigation. The investigation was performed at age 47 years and revealed a slight general brain atrophy that was more pronounced in supratentorial regions (Fig. 1b-c). Cerebellar hemispheres and vermis showed mild atrophy (Fig. 1d-e) whereas the brainstem had normal appearance. The assessment was performed qualitatively by a neuroradiologist (JW) with 25 years of experience and based on literature findings in subjects at different ages, and is considered accurate for clinical assessment of individual patients [8].

### Whole exome sequencing, genetic analysis and 3D protein modeling

The mode of inheritance and the extensive genetic heterogeneity in SCA prompted us to perform whole exome sequencing (WES) on DNA from two affected individuals (ind. IV:2 and IV:3) on the Ion Proton System (Life Technologies, Carlsbad, CA, USA) as described previously [9]. Alignment of reads to the human reference sequence (hg19 assembly) and variant detection was performed using v2.1 of the LifeScope™ Software (Life Technologies, Carlsbad, CA, USA). SNPs and indel data was stored in an in-house exome database together with variant annotation information obtained from ANNOVAR and dbSNP135 [10]. Custom R scripts were used to identify potentially damaging variants that were shared between the patients while not present in any of the other ~2000 exomes in the in-house database.

Segregation analysis on DNA from all seven available family members was performed by bidirectional Sanger sequencing (Applied Biosystems Big Dye Terminator v3.1 Cycle Sequencing Kit, Applied Biosystems, Life Technologies, Carlsbad, CA, USA) on a 3730xl DNA Analyzer (Applied Biosystems, Life Technologies, Carlsbad, CA, USA). Sequencher software (Gene Codes Corporation, Ann Arbor, MI, USA) was used for analysis of Sanger sequencing results. In silico predictions of the effect of gene variants was performed using PolyPhen-2, MutationTaster and PROVEAN [11–13]. The possible effect of the variant on splicing was investigated using BDGP: Splice Site Prediction [14].

**Fig. 2:**
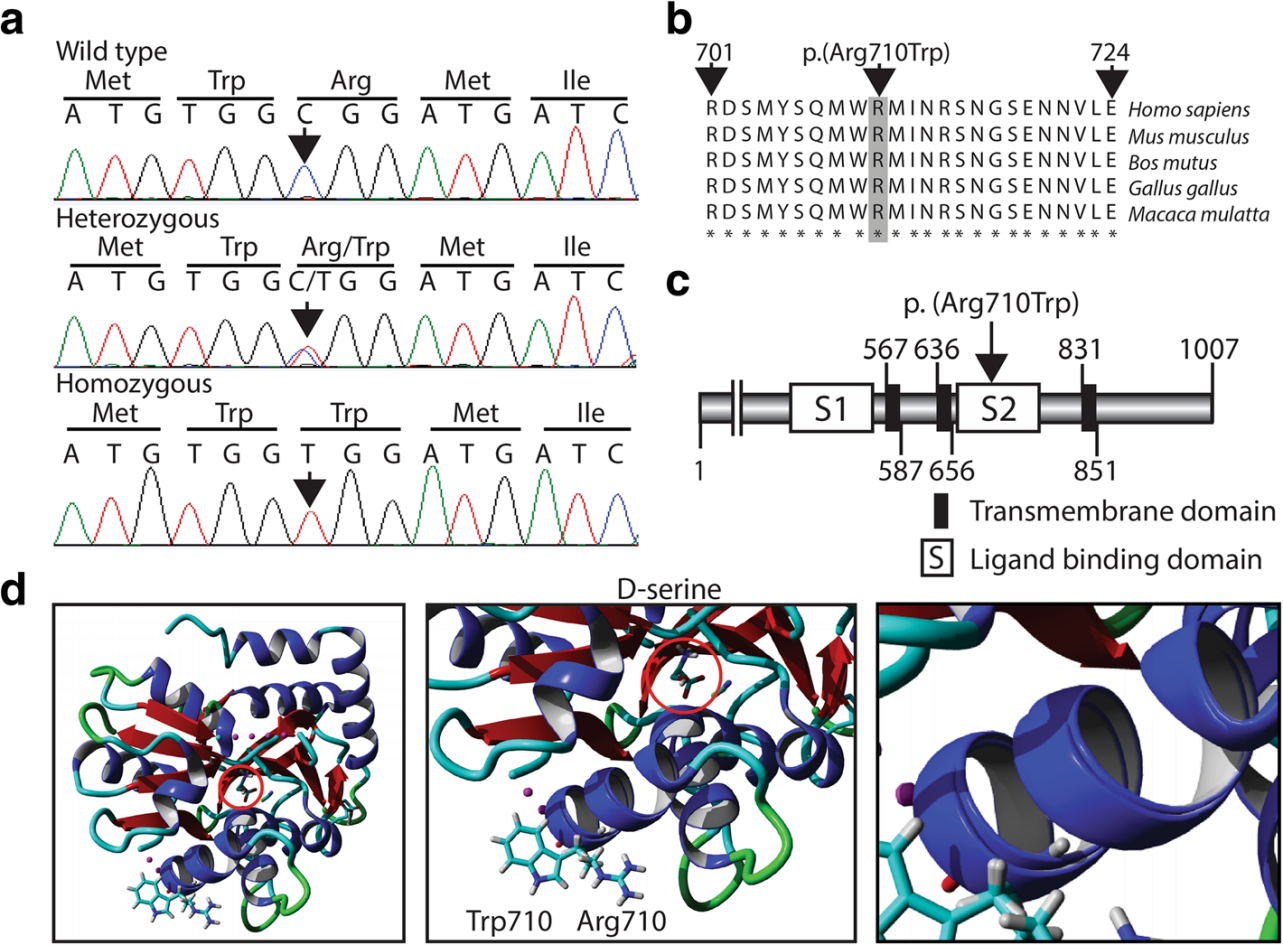
Analysis of the *GRID2* variant and 3D modeling of GluD2. (**a**) Sequence chromatogram of genomic DNA showing part of the *GRID2* gene obtained from the healthy sibling IV:5 (top), the heterozygous mother (middle) and a homozygous affected individual IV:2 (bottom). Arrows indicate the position of the c.2128C > T transition. (**b**) Degree of conservation of the Arg710 residue (shaded, bottom) across different species. (**c**) Relative position of the p.(Arg710Trp) substitution in the second extracellular serine-binding domain of the GRID2 protein. NH_2_: N-terminus; COOH: C-terminus. (**d**) Overview of the 3D structure of GluD2 protein with D-serine circled in red (left box). Enlargement of the D-serine binding domain of GluD2 with juxtaposition of the w.t. (p. Arg710) and the mutated (p.Trp710) residues at position 710 (middle box). The p.Arg710Trp substitution predicts a slight conformational change in the α-helix of the ligand binding domain (right box). The position of D-serine is circled in red

The WES data was filtered for shared homozygous or compound heterozygous variants in the two sequenced individuals. Filtering revealed two homozygous and twelve compound heterozygous non-synonymous single nucleotide variants, including a homozygous c.2128C > T, p.(Arg710Trp) missense variant in the *GRID2* gene. Among the 14 recessive variants, 13 was excluded either by segregation analysis in our family or by being present in homozygous form in the GnomAD database without associated neurological features [15, 16]. Based on the previous association of *GRID2* variants with SCA and the absence of other candidate variants we considered the c.2128C > T variant as a likely genetic cause for the disease. Sanger sequencing revealed that all three affected individuals are homozygous for the variant while two asymptomatic family members (one sibling and the mother) are heterozygous (Figs. 1a and 2a). The resulting amino acid substitution p.(Arg710Trp) alters an evolutionary conserved residue in encoding the extracellular serine-binding domain of the protein (Fig. 2b–d) [17]. The *GRID2* variant was predicted to be damaging by MutationTaster (disease causing), PolyPhen-2 (probably damaging with a score of 1) and PROVEAN (deleterious with a score of −4.39). Furthermore, the variant was excluded in 200 ethnically Pakistani control chromosomes and it is not present in the EVS data release (ESP6500) on the Exome Variant Server, NHLBI GO Exome Sequencing Project (ESP), Seattle, WA or the Exome Aggregation Consortium (ExAC) database, suggesting that the missense mutation is very rare [18, 19]. We reported the *GRID2* variant to the ClinVar database with the submission accession: SCV000502994.1 [20].

Structure of the ligand-binding core of GluD2 in complex with the position of D-serine was retrieved from the protein data base (PDB; rcsb.org/pdb; PDB ID: 2v3u). The relative position of residue 710 in relation to D-serine was visualized using RCSB - Protein Workshop Viewer [21]. The effect of the variant at the protein level was investigated using BuildModel command in FoldX (foldxsuite.crg.eu) together with the YASARA software to introduce the change p.Arg710Trp (corresponding amino acid in the ligand-binding core model is Trp162) [22]. Juxtaposing the structure of the GluD2 protein with p.Trp710 showed that the a.a. substitution predicts a minor conformational change of the ligand binding domain illustrated by a slight shift of the α-helix (Fig. 2d).

## Discussion and conclusions

The GluD2 protein, encoded by *GRID2*, is a member of the ionotropic glutamate receptor family that mediates excitatory synaptic transmission [17]. Studies on mice have revealed that *Grid2* is expressed primarily in the Purkinje cells and it is essential for the formation and organization of synapses [23, 24]. Furthermore, mice with homozygous disruption of *Grid2* show ataxia and mild cerebellar hypoplasia [25]. In humans, a few studies have recently reported on *GRID2* gene variants in cerebellar syndrome with variable clinical expression. Characteristic features include slowly progressive SCA, ocular symptoms including upgaze and nystagmus, hypotonia, developmental delay with cognitive decline, and reduced volume of cerebellar vermis. The symptoms have been associated with both biallelic or monoallelic mutations indicating alternate patterns of inheritance [3–7].

Our combined data show that a novel and homozygous missense variant in the *GRID2* gene is associated with the clinical features in our family. Furthermore, the observations confirm that the gene is essential for normal cerebellar development and function. In addition, results from MRI of one affected individual suggest that the *GRID2* mutation in our family is associated with atrophy of supratentorial brain regions detected as a generalized, moderate widening of cerebral sulci. The supratentorial changes were revealed at adult age and although the intellectual disability appeared stationary from adolescence, it cannot be excluded that the MRI abnormalities reflect a late stage of the disease and in cases with overt cognitive symptoms. Interestingly, the cerebellar abnormalities showed atrophic changes but these were surprisingly mild. Unfortunately, only one out of the three affected siblings were available for MRI and individual variations among the three affected siblings cannot be excluded. Furthermore, all three patients in our study presented with peripheral hypertonia since early childhood as well as normal oculomotor functions and vision. This adds to the features in all previously reported cases with *GRID2* associated cerebellar syndrome.

The clinical variability and the presence of both recessive and dominant inheritance for *GRID2* mutations are puzzling. Interestingly, alternate inheritance patterns have been observed in other neurological disorders, e.g. in SPG7 due to paraplegin mutations [26] and in SPG3 due to atlastin mutations [27]. It may be hypothesized that the variability is caused by the position and structure of the mutation and/or by gene modifiers. All ionotropic glutamate receptor family members (iGluRs), including Glud2, consist of two extracellular domains, an amino-terminal and a ligand binding domain (ATD and LBD). The ATD binds to a Cerebellin-1 Precursor (Cbln1) hexamer, thereby anchoring Glud2 to beta-neurexin (β-NRX1). This results in a conformational change and allows for binding of the ligand D-serine that leads to an opening of the ion channel [28]. The activation of GluD2 by D-serine regulates long-term depression (LTD) at synapses between parallel fibers and Purkinje cells in the immature cerebellum [29]. The missense variant in *GRID2* in our study alters a highly conserved residue in the D-serine binding site of GluD2 predicted to be damaging. No missense mutations have previously been reported within this domain of the GluD2 protein. Thus, it cannot be excluded the atypical clinical features associated with homozygosity for the *GRID2* variant reported herein are caused by specific effects on synaptic transmission. The fact that the two heterozygous carriers for the gene variant present without any neurological or cognitive symptoms supports autosomal recessive inheritance and suggests an additive effect of two mutated alleles in our family. The GluD2 residue 710 is situated in the D-serine binding domain but outside the ligand interacting region. Furthermore, the amino acid change predicts a minor conformational change of the domain detected in the α-helix (Fig. 2d). These observations are consistent with altered, but not abolished D-serine binding properties of GluD2 and may require both mutated alleles for a phenotypic expression [29]. However, the precise effect of the *GRID2* variant c.2128C > T on GluD2 function will require further analysis e.g. by expressing the mutated allele in model systems.

The SCAs comprise a large number of clinically and genetically heterogeneous disorders with all possible inheritance patterns. This constitutes a diagnostic challenge further complicated by clinical overlaps between the different clinical entities. In this context, WES has evolved as an important diagnostic tool, especially when clinical information is incomplete or when patients are unavailable for investigations [30]. In our family, the diagnosis was predicted from the exome sequencing results and later confirmed by a thorough follow-up with a clinical neurological examination and MRI that showed mild general cerebral and cerebellar atrophy, increased peripheral muscle tone, absent ocular symptoms and neurodevelopmental delay.

In conclusion, we identified a novel homozygous *GRID2* missense mutation [c.2128C > T, p.(Arg710Trp)] segregating an autosomal recessive cerebellar syndrome in three siblings. The missense variant is located in a conserved region encoding the extracellular serine-binding domain of GluD2 that predicts a change in protein conformation from 3D modeling. The clinical presentation is atypical for *GRID2* associated ataxia and our patients present with widespread supratentorial brain abnormalities, absence of oculomotor symptoms and increased peripheral muscle tone. Additional features include slowly progressive cerebellar ataxia and delayed psychomotor development. Our combined findings add to the clinical and genetic variability associated with *GRID2* variants highlighting the complexity of cerebellar syndromes. We further demonstrate the usefulness of WES as a valuable diagnostic tool when combined with deep phenotyping in cases with an unspecific and complex form of ataxia.
